# NTP Nonneoplastic Lesion Atlas: A New Tool for Toxicologic Pathology

**DOI:** 10.1289/ehp.122-A76

**Published:** 2014-03-01

**Authors:** Charles W. Schmidt

**Affiliations:** **Charles W. Schmidt**, MS, an award-winning science writer from Portland, ME, has written for *Discover Magazine*, *Science*, and *Nature Medicine*.

When the National Toxicology Program (NTP)^^1^^ was launched in 1978, its pathologists were tasked mainly with screening environmental chemicals for cancer in rodents. Peering through microscopes at brightly illuminated tissue slides, they became adept at finding and categorizing tumors, or “lumps and bumps” as they’re also called in the pathologist’s trade. Those pathology readings have formed the basis for countless health-protective standards for chemicals in food, water, and air.

Since the launch of the NTP, noncancer diseases linked to environmental factors have also drawn mounting concerns. Heart disease, for instance, tops cancer as the nation’s leading killer, now accounting for about 1 in 3 U.S. deaths.^^2^^ With the environmental spotlight shining brighter on noncancer illnesses, toxicologic pathology is evolving. A new realm of so-called nonneoplastic lesions, or tissue changes that are pathological but not cancerous, has become a growing priority for the field.

**Figure:**
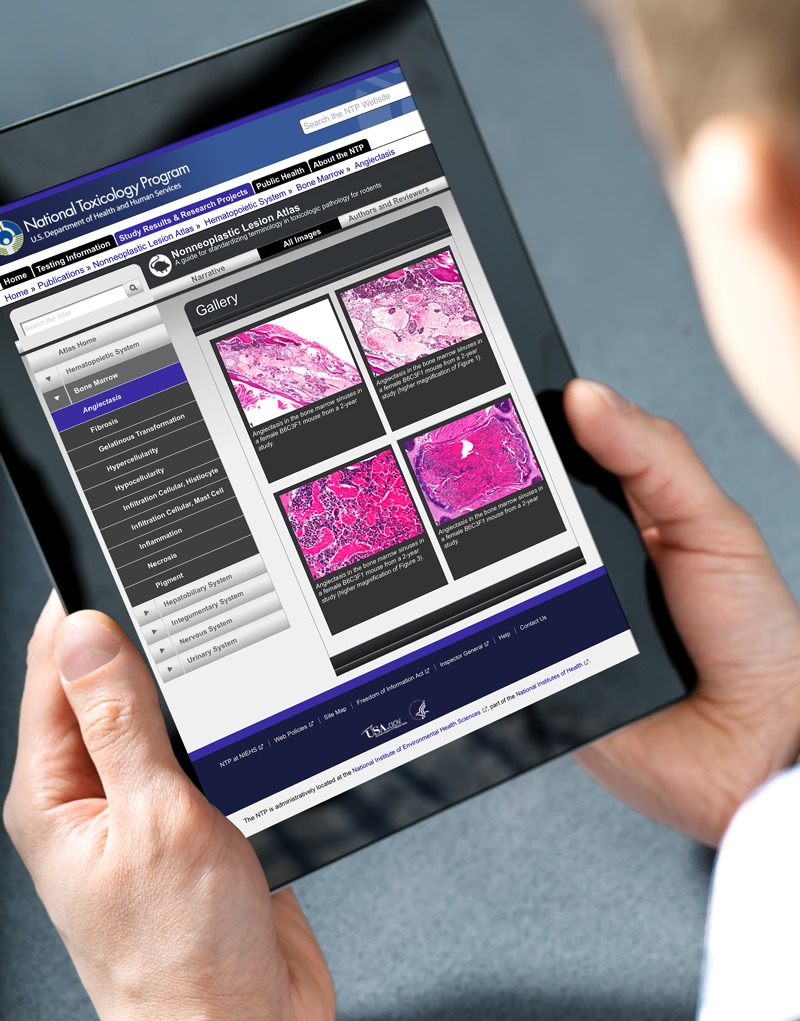
A new online atlas of images and diagnostic guidance promises to significantly improve researchers’ ability to evaluate noncancer findings in animal studies. © Shutterstock; Joseph Tart

For decades, pathologists have diagnosed nonneoplastic lesions and other tissue findings by matching what they see under the microscope with a picture in a textbook or journal. Now, a recently launched website hosted by the NTP is bringing that process into the digital age. The NTP Nonneoplastic Lesion Atlas, unveiled 30 January 2014, promises to significantly improve the ability of researchers to evaluate noncancer findings in animal studies.

The Atlas was developed over more than five years, during which time NTP pathologists and their collaborators pored over lesion images in a mission to define precisely what they should called. It provides consensus guidelines for diagnosing nonneoplastic lesions observed in NTP studies^^3^^ and thus will help resolve discrepancies over how lesions are characterized and described in the NTP’s database. The guidelines “provide recommendations that pathologists can use to refer to the lesions in the same way,” explains NTP associate director John Bucher.

The new site will also enable researchers to compare studies, search the NTP database more effectively, and generate control data. “It’s a heavily illustrated online resource that we can use as a teaching tool and a reference for anyone who reads our studies,” Bucher says. The same lesion may be described using different terms by different pathologists, adds Gary Boorman, a veterinary pathologist with Covance Laboratories in Chantilly, Virginia, “so a goal of the Atlas is to encourage pathologists to use the same terminology and grading systems.”

## A Growing Focus on Nonneoplastic End Points

Derived from Greek, the term “neoplasia” literally means “new plasma” and refers to new growth in tissue that does not serve a useful purpose—i.e., tumors. Neoplasms may be malignant or benign; some benign tumors may progress to malignancy.

According to Boorman, coeditor of a classic text in the field,^^4^^ nonneoplastic lesions encompass a very broad assortment of tissue alterations including congenital, degenerative, inflammatory, adaptive, and reparative changes. Some nonneoplastic lesions occur normally with age; exposure to a test chemical may either increase or decrease the incidence and/or severity of these spontaneously occurring “background” lesions. In other cases, exposures may induce novel nonneoplastic changes. Some nonneoplastic lesions may progress to tumor formation with time or continued chemical exposure.

Pathologists have traditionally diagnosed tissue lesions using print resources, such as textbooks and journals. With the Atlas, users are able to access and zoom in on hundreds (and eventually thousands) of high-resolution images online. Each lesion is accompanied by commentary and recommendations for accurate diagnosis, supported by peer-reviewed references.

NTP pathologist Mark Cesta, who serves as the Atlas’ primary editor, says NTP staff and their collaborators strove for consistency with established texts in the field, notably *Pathology of the Fischer Rat: References and Atlas*^^3^^ and *Pathology of the Mouse: References and Atlas.*^^5^^ Notably, the NTP Atlas includes slides derived from studies with the Sprague-Dawley rat, which is now more widely used in NTP research than its predecessor, the Fischer 344 rat. Veterinary pathologists rely increasingly on the Sprague-Dawley rat because Fischer 344 rats are at naturally high risk of mononuclear cell leukemia and testicular tumors,^^6^^^^,^^^^7^^ which can muddy study results. According to Boorman, these two strains have been used more than any other in product safety assessment and evaluation of environmental chemicals.

The Atlas is also consistent with documents issued by the International Harmonization of Nomenclature and Diagnostic Criteria (INHAND), a global toxicologic pathology initiative to develop consensus terms for diagnosing lesions in rats and mice.^^8^^ Launched in 2006, INHAND publishes its recommendations in the peer-reviewed journals *Toxicologic Pathology* and *Journal of Toxicologic Pathology*, dedicating separate supplemental issues to specific organ systems.^^9^^ By contrast, the Atlas’ content resides in a single online location.

Whereas INHAND is geared toward scientists in the pharmaceutical industry and limits its content to diagnostic terminology, the Atlas was developed mainly as an internal resource for the NTP with an additional focus on strategies for consistency in diagnosing lesions. But with the NTP’s far-reaching influence in toxicologic pathology, Boorman predicts the Atlas, which anyone may use for free, will find widespread use in training and research.

## Diagnostic Challenges

The fact that veterinary pathologists generally agree on neoplastic terms reflects a long focus on cancer in animal studies. Neoplasms are distinct, easily recognizable entities, unlike nonneoplastic lesions, which raise more diagnostic challenges. For instance, a chemical that induces inflammation as its primary effect might also induce secondary changes, such as metaplasia (an adaptive shift from one cell type to another) or necrosis (tissue death).^^10^^ But given that inflammation can induce necrosis and vice versa, it’s not always obvious which lesion came first. Moreover, inflammation can take different forms—for instance, acute, suppurative, or chronic—and there may be considerable overlap between these subclassifications. “The same inflammatory lesion might be subclassified differently depending on who reads the slide,” Cesta says.

In yet another complicating factor, many nonneoplastic lesions occur normally with age but become more pronounced with chemical exposure. Thus, it can be difficult to discern chemical effects from a lesion’s natural background rate. One example, Boorman explains, is a kidney disease called chronic progressive nephropathy (CPN), which occurs to some degree in all rats as they age. CPN displays multiple features on histology, including an influx of white blood cells, an accumulation of connective tissue, and a buildup of calcium and phosphate in the kidney. Depending on the pathologist’s interpretation, each of these findings might be recorded separately, or they might be lumped together as CPN. Therefore, a search for “CPN” in the NTP database might not return a complete set of results for studies that report CPN characteristics.

In a related scenario, Boorman says that aging female Fischer 344 rats normally accumulate high levels of basophilic foci in the liver, meaning small clusters of liver cells that stain a darker blue than the normal liver, indicating they might be precancerous. These age-related lesions don’t all turn into tumors. Yet, nitrosamines, which are used to manufacture certain products and also occur as by-products, produce basophilic foci that do often become malignant.

“In treated animals, the foci precede cancer, but in control animals, they are very unlikely to progress to cancer,” Boorman says. “So that’s a case in which the same nonneoplastic term—‘basophilic foci’—could apply to very different scenarios.” He says that discrepancy could be a challenge for pathologists trying to compare the dose-related incidence of basophilic foci in chemically treated animals with background lesions in untreated controls.

Many nonneoplastic diseases are associated with environmental exposures, and lesions that appear in human illness can also be observed in chemically treated animals. For example, when exposed by inhalation to a chemical called diacetyl (an ingredient in artificial butter flavoring), C57BL/6 mice develop bronchial lesions similar to a human illness known as bronchiolitis obliterans. This irreversible obstructive lung disease has been known to occur in workers with occupational exposure to artificial butter flavoring.^^11^^ Ideally, by improving how pathologists classify and interpret these and other nonneoplastic lesions, the Atlas will enhance our understanding of environmentally related diseases in humans.

Meanwhile, Bucher adds that discrepancies in terminology create a huge workload for the NTP’s Pathology Working Group, which is responsible for resolving differences of opinion on diagnostic issues. Pathologists have to read reports and compare images that can be described differently depending on the source as they try to come up with an accurate diagnosis. The task can be onerous, “and this is a problem that’s just getting worse as we get farther into noncancer end points,” Bucher says. “We’re doing more and more work in reproductive, immunological, neurological, and developmental toxicology, and that means more time analyzing nonneoplastic lesions.”

According to Robert Sills, chief of the NTP Cell and Molecular Pathology Branch, nonneoplastic lesions tend to occur soon after chemical exposures, unlike tumors, which can take much longer to appear. The Atlas will provide guidance for achieving greater consistency in both short- and long-term studies, Sills says.

## From Systems to Lesions

To access the Atlas’ content, users navigate from a homepage organized around anatomical systems.^^3^^ At the time of this publication, 5 systems had been published online: the hematopoietic system (which makes blood), the hepatobiliary system (i.e., the liver and gallbladder), the integumentary system (i.e., skin), the nervous system, and the urinary system.

Each system is subdivided into pages for relevant organs and tissues. The nervous system, for instance, has pages devoted to the brain, nerves, and spinal cord. In turn, the page for the brain has an illustrated anatomical discussion and 18 additional pages, each devoted to a specific brain lesion. The pages include multiple enlargeable images of the lesions along with the diagnostic recommendations.

According to Cesta, the Atlas will eventually be expanded to 13 anatomical systems encompassing 56 different tissue types in all. “We think of it as a living document that will be continually updated,” he says. Since the Atlas is a living document, pathologists will have the flexibility to diagnose new lesions as they emerge in research, and the Atlas can incorporate new terms for novel findings.

Much of the initial NTP research and pathology review is performed by contractors, who will be encouraged to use the consensus terms contained in the Atlas. “NTP pathologists review all the work that our contractors do for us,” Sills says. “We meet with our contractor partners one on one, and that gives us the opportunity to confirm that our recommendations for documenting nonneoplastic lesions are being followed.”

Prior to its official launch, the Atlas underwent extensive review coordinated primarily by Cesta, Sills, NTP colleagues David Malarkey and Ronald Herbert, and Amy Brix of Experimental Pathology Laboratories, Inc., in Research Triangle Park, North Carolina. Additional review was conducted by outside experts. Among them was Rick Hailey, a veterinary pathologist with GlaxoSmithKline in Research Triangle Park. Hailey says there’s little difference between drug-induced lesions and those induced by environmental exposures, indicating that pharmaceutical scientists may find the Atlas useful. “That’s especially true of younger scientists,” he says, “who might gravitate naturally to a web-based application instead of the more traditional textbooks.”

Hailey says he views the Atlas as a valuable supplement to INHAND, “primarily for folks that evaluate NTP studies and younger students in training.” He adds, “It’s an intuitive program and a valuable search tool. From the standpoint of functionality, it works well.”
